# The 1999 international emergency humanitarian evacuation of the Kosovars to Canada: A qualitative study of service providers' perspectives at the international, national and local levels

**DOI:** 10.1186/1475-9276-4-1

**Published:** 2005-01-12

**Authors:** Nancy Fowler, Lynda Redwood-Campbell, Elizabeth Molinaro, Michelle Howard, Janusz Kaczorowski, Morteza Jafarpour, Susan Robinson

**Affiliations:** 1 Department of Family Medicine, McMaster University, 1200 Main Street West Room HSC 2V9, Hamilton Ontario L8N 3Z5 Canada; 2 Settlement and Integration Services Organization (SISO), 360 James Street North LIUNA Station-Lower Concourse Hamilton Ontario L8L 1H5 Canada

## Abstract

**Background:**

In response to the Kosovo crisis, Canada received 5,500 Albanian Kosovar refugees in 1999 as part of the emergency humanitarian evacuation and settlement effort. This study attempts to describe the experiences of service providers at the international, national, and local levels, involved in the organization and delivery of health and settlement services in Canada for the Kosovar refugees.

**Methods:**

A qualitative case study design using key informant interviews was used. Nominated sampling was used to identify 17 individuals involved in the organization and delivery of health and settlement. Key themes were identified and recommendations made to provide a framework for the development of policy to guide response to future humanitarian emergencies.

**Results:**

Six themes emerged: (1) A sense of being overwhelmed, (2) A multitude of health issues, (3) critical challenges in providing health care, (4) access to health and settlement services, (5) overall successes and (6) need for a coordinated approach to migration health.

**Conclusions:**

For those involved, the experience was overwhelming but rewarding. Interviewees' major concerns were the need for a more comprehensive and coordinated approach to the flow of medical information and handling of specific health problems.

## Background

The Kosovo crisis resulted in the largest population displacement in Europe since World War II. During this time, over 300,000 Albanian Kosovars were expelled from Kosovo, [1] resulting in a Complex Humanitarian Emergency. (CHE). The United Nations High Commission for Refugees (UNHCR) managed a Humanitarian Evacuation Program (HEP) to move the Kosovars to new international destinations, including Canada.

Canada received approximately 5,500 refugees in 1999, with approximately 500 arriving to the city of Hamilton, in southern Ontario. Some Kosovars stayed temporarily in military bases and arrived after sponsorship housing had been arranged, while others arrived directly to temporary reception "houses" established at two Hamilton hotels. The reception in Hamilton included the organized provision of health services by physicians, nurses, dentists and optometrists, and settlement services from the Settlement and Integration Services Organization (SISO).

This HEP was unique for many countries, including Canada, and involved a complex coordinated effort on the part of international, national, and local organizations traditionally involved with immigrants and refugees, settlement, and health. This event was an important opportunity for health and settlement organizations operating at international, national, and local levels to critically examine and reflect on their efforts to organize and deliver health and settlement services to new immigrants and refugees.

The goal of this study was to explore the main challenges and successes of the Kosovar arrival, from international, national, and local perspectives and to develop recommendations to advise and guide planning of the future complex humanitarian emergencies, using interviews with key informants.

## Methods

A qualitative case study methodology was used with semi-structured interviews of key informants who were involved in the humanitarian evacuation at the international, national and local levels in Hamilton, Canada. This method was deemed most appropriate to provide a framework for understanding the challenges and successes associated with Complex Humanitarian Emergencies and generate recommendations for future efforts. The background of research team members was diverse. Medicine, nursing, anthropology, sociology and settlement were represented. Some members had been directly involved with the Kosovar settlement process at the local level (NF, SR, MJ).

### Sampling

A purposive sampling strategy was used, in which appropriate key informants known to the researchers were approached for participation first. Snowball sampling, whereby additional participants were identified by the initial respondents, was used to increase the diversity of respondents. These sampling approaches are used when certain known individuals are likely to have in-depth information on a topic [2]. Individuals from agencies involved with the 1999 Kosovar HEP were included. There was a very specific attempt to sample individuals from the international to the local levels even if they had no direct association with the Hamilton group. The sampling included three Health Canada officials who were known to be involved at the international and national levels and two Citizenship and Immigration Canada (CIC) officials involved at the provincial and local levels. Also sampled were two representatives from the Department of Social and Public Health Services in Hamilton, five local healthcare professionals (one family physician, two nurse practitioners, one dentist, one optometrist), and five local settlement providers. All of the CIC and the local key informants were referred and identified by the local settlement agency SISO as groups that had been integral to the Kosovo efforts in Hamilton. The international and national key informants were identified through Health Canada and internationally as people who were involved in the effort. An initial group of nine key informants identified eight additional participants. All 17 key informants approached, agreed to participate.

### Interviews

Interviews were conducted in English between April and July 2001. The interviewer (EM) took extensive field notes to supplement the taped and transcribed interviews. Of the 17 key informant interviews, the three international and national ones were conducted by telephone, and 14 were in person at the workplace of the interviewee. Six participants were male and 11 were female. Interviews were approximately 30 minutes in length. The interviews were based on an interview guide and the main part of the interview focused on the organization and delivery of health services to the Kosovars. Questions were further refined during the study. Specific probes were used to follow up on open-ended questions, where appropriate.

Consultation with the local settlement agency (SISO) took place throughout the research process. Key informants were told that the purpose of the study was to gather information that would help in planning for similar events in the future. All participants provided verbal consent and were assured of confidentiality of responses. All interviews were tape recorded with the participants' permission and all but one (due to technical difficulty) was transcribed. Ethics approval for the study was received from McMaster University Research Ethics Board.

Questions pertained to the participants' involvement in the process, challenges, flow of information and communication, and involvement in health care services and settlement.

### Data Analysis

Two research team members (EM and NF) initially independently reviewed the transcripts, coded categories and themes, and then compared results. A third researcher with expertise in qualitative research, who was not involved in the project, also reviewed the transcripts using NVivo software version 2.0 (QSR International Pty Ltd, Melbourne, Australia) and compared results. The interviewer's field notes were also used for comparison with the data. Ambiguities were resolved and themes were developed from categories through discussion among the research group members and re-reading of transcripts. This process continued until no new themes emerged from the interviews. It was felt that saturation was achieved after 17 interviews, and data collection was ended. The process was iterative, thus the later interviews probed new emerging themes identified in previous interviews.

## Results

Six core themes emerged from the data analysis.

### Theme 1: A Sense of Being Overwhelmed

There was agreement among the participants that the Kosovo crisis in 1999 was very unusual in nature. Participants stressed that there was a lot of "scrambling" to prepare the infrastructure to receive a large number of people in a very short period of time. Respondents/informants were surprised by the magnitude, immediacy, scope and scale of the response required. Most described feeling overwhelmed.

Although the local settlement agency (SISO) had begun preparations by meeting with various local agencies and organizations weeks in advance, they were given only three days notice by national immigration authorities that the Kosovars were arriving locally. One Hamilton Social and Public Health Services representative noted "We had many community people come together to talk about how we could plan for this big influx of refugees... we knew were in dire straits". One settlement worker called it "organized chaos". One local health care professional explained the triage process and it was evident that a major organizational effort was required to process the arrivals.

"We had to do our own triage at the hotel to find out whether there was anybody needing medications, anyone with heart conditions, anyone with diabetes ... we had to scan the place to find out, and even then we didn't have medical records that came with them... We just went around asking: Do you know about anybody who is pregnant? Do you know about anybody who has heart conditions? Do you know about anybody who is diabetic? ... And we tried to identify those people or they presented in the little clinic".

### Theme 2: A Multitude of Health Issues

Participants described facing a multitude of challenges in responding to the health needs of Kosovar refugees. Women's health services, including pregnancy care and abortion services were required. Dental care, mental health services and general curative services were also required. Participants were struck by the poor oral health of the Kosovars. Many Kosovars had been deprived of any curative or preventative care in Kosovo for a number of years prior to the 1999 crisis. One local health care professional observed,

"I noticed with the Kosovars...that a lot of them had not had any health care for a long time so they had many of the things that we take for granted. Immunizations being up to date and those sorts of things had not been done.... They only saw doctors if they were absolutely on their deathbed. So sometimes, you sort of had a lot of catching up to do in terms of getting their health up to date"

Chronic and poorly managed conditions such as diabetes, hypertension and renal failure were common. An interviewee with an international perspective noted,

"I think the world, from a health point of view, went into Kosovo thinking that all refugees were like the Great Lakes and Rwanda...The challenges [in Kosovo] were more of chronic diseases, diseases of socialization, hypertension, diabetes, renal failure...I think that people have learned to be a bit more comprehensive in their approach to conflicts and emergencies."

### Theme 3: Critical Challenges in Providing Health Care – Lack of information at the local level

The participants expressed three main challenges which made their work more difficult: (i) tuberculosis screening, (ii) the lack of medical records and tuberculosis test results and (iii) mental health issues.

Several participants noted that the tuberculosis screening process was not optimally organized. One local healthcare professional lamented,

"I was reassured, but without any documentation to back it, that everyone had been screened for TB...When I contacted Public Health, they had not received any notification ...Certainly I didn't know where or how to get that information, and it seemed to me that Public Health didn't either".

Communication to local health care providers about test results (tuberculosis and radiographs) was often described as sub-optimal. Immigration officials in Europe attempted to keep medical information flowing. One interviewee from an international perspective explained that,

"We provided by fax and e-mail a summary of the medical conditions on the aircraft, so that they can be dealt with appropriately on arrival in Canada...We also used colour-coded cards and things so that (when) people who got off the plane (who needed care) they did receive expedient care".

However, many local respondents expressed concern over the location of medical records and the inability to access this information in a timely manner. A local healthcare professional expressed,

"At no point did I receive any medical documents about people with serious or chronic medical conditions that required care...if that information was available, I never got it".

It was clear among the participants that mental health concerns were prevalent and service providers struggled with the delivery of appropriate mental health services. One local healthcare professional explained,

"...things like headaches presenting when really the underlying condition was one of stress and anxiety, distress. Most of them came under the umbrella of what we might call, presenting with trivial complaints, but really what was beneath it all was stress and anxiety..".

### Theme 4: Access to Health and Settlement Services

The rapid settlement of the Kosovars resulted in the local collaboration and coordination of health and settlement services at a single geographic site. This multi-agency collaboration was thought to enhance access and provision of services. One local healthcare professional described,

"[The Kosovars] had, I thought, very well organized access...The SISO organization provided chauffeurs and translators, and administrators to pull all those areas together. So...if a refugee needed to go to a lab, they were driven, they were translated for, and they were brought back...".

Since translators organized through SISO were available at the local health care sites, language was not perceived as a significant challenge by local health care providers.

There was an awareness that the care provided was transitional in nature. This created some hesitancy in initiating treatment, as frequently there was uncertainty about the medical follow-up arrangements. Mental health issues and the lack of opportunity for identification and communication with future care providers were identified as concerns.

While the Kosovars received medical coverage through the Interim Federal Health (IFH) program, local health care providers felt that the amount and nature of coverage was inadequate for services such as home care, dental care and optometry. One local healthcare professional described the issues surrounding home care,

"One access issue that came up was the fact that under their IFH coverage, IFH does not cover home care, and without special arrangements to be in place...they don't qualify for it...There were a lot of people...who had walked miles and miles...and I saw [some] who had really bad foot conditions, infections and ulcers requiring daily treatment, soakings, dressings, bandage changes... Normally, those are things in the community that we would involve home care in...".

In several instances, both the optometrist and dentist interviewed provided services that were not covered, free of charge, but in general, there was much confusion and uncertainty regarding the payment scheme for professionals. A dentist explained,

"..we were not informed as a health professional that if we were approached by people from Kosovo that plans were available for treatment...we knew absolutely nothing until we started asking the questions..".

### Theme 5: Overall successes

Specific examples of successes included provision of comprehensive onsite health care integrating both health and settlement services, the use of nurse practitioners to allow physicians to focus on more complex cases, the policy to keep families intact, and the positive media coverage that contributed to an atmosphere of acceptance. A local settlement provider observed,

"The Kosovars was something that the whole nation took on...We saw what kind of role the media can play in making the host community aware what other people are going through, explaining that refugees are not to be seen as invaders but as people who are in need of welcoming, and the Kosovars received one of the best, I think at least in my experience, one of the warmest welcomes...".

Services available to the Kosovar refugees were deemed better compared to that provided to other refugee groups. This was a concern to many participants. Some stated that all refugees should be offered the same standard of high quality settlement services as those made available to the Kosovars. Another local settlement provider noted,

"Refugees, regardless from where, should be treated the same way because it creates resentment not just in other refugee communities that have come to Canada, but [it] creates resentment among the workers that are providing services and sometimes struggling to get resources for a group of people...The Kosovars should be an example of how refugees should be treated in general".

### Theme 6: Need for a Coordinated Approach in Migration Health

A number of participants suggested that we should learn from this experience with the Kosovars and prepare to put a contingency plan in place for similar events in the future.

Better communication and organization were repeatedly stressed. One participant used the term 'Migration Health' to describe such an overall coordinated approach. Several participants commented on the importance of the coordinated approach as a necessary societal investment. One interviewee from an international perspective noted,

...a lot of people who are working on the receiving end are simply following the process for receiving refugees.... there may not be the resources to look at the longer term issues: primary health care, health education, explaining how the health systems work, looking at some of the parameters that may influence longer term mortality and morbidity: dietary counselling, smoking cessation counselling, primary preventive health care procedures that we do in Canada that may not take place in the developing world."

Most participants suggested that health care and settlement providers need to enhance their cultural sensitivity and cultural competence and better understand the health conditions of the displaced individuals in their country of origin.

## Discussion

Despite organized attempts to coordinate efforts at different stages of the migration process, communication gaps and the sheer size of the influx resulted in challenges at the local level. Officials and service organizers at the international/national levels were unaware of these local gaps at the time of the evacuation. The flow of medical information and health records is an example. Primary care health workers needed to have easy access to targeted health information about the Kosovars, however this information was not available. If this information had been more easily available, health care services may have been more streamlined, and unnecessary duplication of lab tests and radiographs could have been avoided.

Health care providers did not know what to 'expect'. Mental health, dental care and communicable diseases (specifically tuberculosis) were identified as requiring further specialized planning. This finding is similar to other refugee experiences in Australia [3] and Canada [4]. Our study was also consistent with other refugee literature suggesting that there is often gap in addressing refugee women's health services [5-7].

There was a consensus among participants that this international evacuation represented an improved approach and a good foundation on which to organize refugee health and settlement in the future. Informants' concerns about local preparedness and the need for future advanced planning was consistent with recent United Nations High Commission for Refugees (UNHCR) findings [8]. Participants wanted to see a contingency plan developed for the future with enhanced communication and better organization. These wishes have been incorporated into the detailed recommendations.

A potential limitation of this study is the time elapsed between the refugees' arrival and the interviews. Asking people to recall events that took place approximately 18 months previously may have influenced the nature and detail of the information obtained. However, it may also have enabled informants to recover from the initial emotional reaction and to see these events from a different perspective.

It must be stated that the 'success' as defined by key informants may be very different than 'success' from the refugees' perspective. This study examined the perceptions of those involved with the Kosovars. The majority (but not all) of the local health professionals and settlement workers interviewed had worked in the local 'settlement houses' site where there was a shorter notice of arrival of the refugees. This may have contributed to the sense of 'chaos' echoed in many of the comments.

Many of the participants in this study made observations about the diverse nature of complex emergencies. The importance of logistics and planning has also been described in other studies [8-10]. Some countries and jurisdictions are starting to develop 'rapid response' protocols for similar situations. After the Kosovar influx, Australia developed a surveillance, triage, clinical and database system (*'Operation Safe Haven'*). Triage questionnaires for primary health care based on International guidelines were developed [11]. *Operation Safe Haven *produced a template for refugee "acute health response" system. Using the Australian template combined with findings from this study, we propose the following attributes for an *International Rapid Response System *(IRRS):

1. Establish lead organizations at the different levels (international, national, provincial, local)

2. Clearly define roles for different organizations involved

3. Establish communication linkages between lead organizations at different levels

4. Identify strategies for flow of information ("situation reports") from authorities to local organizations

5. Establish a protocol for triage/rapid assessment of health, settlement needs and cultural preferences

6. Establish a system of medical charts that follow individual refugees though the process

7. Establish links to primary and specialized health care especially for urgent communicable disease and mental health issues

8. Identify a plan for the provision of urgent dental and eye care

9. Establish a surveillance/data-tracking system to collect essential health information, tack service use, and provide the ability to conduct quality assurance assessments

10. Use information technology – key internet links; background data; briefing; high quality background and cultural information about refugee groups

11. Introduce better training of professionals who will be dealing with refugees. Includes increasing cultural competence of health care and settlement providers, women's health, mental health, chronic illness, dental care and current health coverage for refugees. Global health issues should be introduced into health school curricula.

12. Local health care professionals need access to better information regarding the background, circumstances and organizational arrangements relating to refugees. Regular, ongoing information sharing sessions with health professionals involved with refugees, public health and government would facilitate communication when the next refugee crisis occurs. There must be political commitment at all levels.

## Conclusions

For those involved in the Kosovar Humanitarian Evacuation Program, the experience was both overwhelming and rewarding. Many perceived that a superior effort was made for the Kosovars compared to other groups of refugees and that positive media coverage contributed to a warm and effectively organized reception. Interviewees' major concerns were the need for a more comprehensive and coordinated approach to the flow of information and handling of specific health problems.

## List of Abbreviations Used

Complex Humanitarian Emergency: CHE

United Nations High Commission for Refugees: UNHCR

Humanitarian Evacuation Program: HEP

Settlement and Integration Services Organization: SISO

Tuberculosis: TB

Interim Federal Health: IFH

International Rapid Response System: IRRS

## Competing Interests

The author(s) declare that they have no competing interests.

## Authors' Contributions

NF and LRC designed and implemented the study, analysed the data and critically revised the manuscript. SR, MJ, MH, JK, SR critically revised the manuscript, EM conducted the interviews, assisted with study design and implementation, data analysis, and drafted the manuscript.
